# Scabies increasing incidence in Bologna from 2013 to 2024: a retrospective analysis

**DOI:** 10.1136/sextrans-2024-056436

**Published:** 2025-01-29

**Authors:** Corrado Zengarini, Martina Mussi, Michelangelo La Placa, Alessandro Pileri, Anna Lucia Virdi, Marco Chessa, Federico Bardazzi, Carlotta Gurioli, Michela Starace, Valeria Gaspari, Cosimo Misciali, Fortunato Cassalia, Bianca Maria Piraccini, Iria Neri

**Affiliations:** 1 Department of Medical and Surgical Sciences, University of Bologna, Bologna, Italy; 2 Dermatology Unit, IRCCS Azienda Ospedaliero-Universitaria di Bologna, Bologna, Emilia-Romagna, Italy; 3 Department of Medicine, University of Padua, Padova, Veneto, Italy

**Keywords:** SCABIES, Epidemiology, SURVEILLANCE, Social Sciences

## Abstract

**Abstract:**

**Objectives:**

Scabies infestation, caused by the *Sarcoptes scabiei* mite, has recently emerged as a public health concern in Western nations, with increased incidence worldwide. In Bologna, Italy, local health authorities report a rise in scabies diagnoses, although detailed data are limited. This study aimed to analyse the temporal trends of scabies cases diagnosed at S. Orsola Hospital’s Dermatological Emergency Department, focusing on significant changes in incidence and seasonal variation over time.

**Methods:**

A retrospective observational study was conducted using data from October 2013 to September 2024, extracted from hospital records using ICD-9 (International Classification of Diseases, Ninth Revision) codes. Variables included monthly case counts, discharge date, patient age and nationality. All pruritic cases from the emergency department were evaluated in the dermatology unit, with diagnosis confirmed via dermoscopic or microscopic examination. Only first visits were included, excluding follow-up visits or post-therapy controls to avoid duplication. Monthly cases were aggregated to identify annual and seasonal trends. χ^2^ tests assessed nationality distribution differences, and linear regression analysed annual trends. Seasonal variation was evaluated with the Kruskal-Wallis test.

**Results:**

A total of 1192 cases were diagnosed. The nationality distribution remained stable, with no significant differences between Italian-born and other nationalities. A significant upward trend in incidence was observed in recent years, with seasonal variation showing higher case counts in February, March and April, and the lowest in July and August.

**Conclusions:**

The increase in scabies cases in recent years and distinct seasonal peaks suggests that environmental and social factors may contribute to transmission in Bologna. Without demographic changes and known drug resistance, factors such as the rise in tourism and suboptimal accommodation conditions may play a role in transmission. Enhanced public health monitoring, awareness and targeted interventions are recommended to manage this trend effectively.

**WHAT IS ALREADY KNOWN ON THIS TOPIC**

- Scabies, caused by the *Sarcoptes scabiei* mite, is a highly contagious skin infestation that has recently re-emerged as a public health concern in many regions, especially in Western nations. Seasonal peaks in scabies transmission have been documented, typically during colder months when indoor crowding increases and is related to travellers and migrant fluxes. However, while there are reports from healthcare providers of rising scabies diagnoses, comprehensive, standardised data documenting long-term trends are often limited.

**WHAT THIS STUDY ADDS**

- This study provides a detailed analysis of scabies incidence trends over a decade in Bologna, Italy, revealing a significant, periodic increase in cases and marked seasonal variation. By confirming that cases peaked in the last years and are aligned with colder months, this study adds valuable evidence to support the influence of seasonality on scabies transmission and discusses the other possible concurrent factors, specifically the impact of overtourism and related living conditions even in the absence of changes in demographic factors or known drug resistance.

**HOW THIS STUDY MIGHT AFFECT RESEARCH, PRACTICE OR POLICY**

- These findings underscore the need for public health authorities to integrate continuous monitoring of scabies cases and develop targeted prevention and treatment strategies, especially during peak transmission seasons. Policymakers may consider addressing the role of housing standards in tourist accommodations as part of scabies control measures. The study also emphasises the importance of timely diagnosis and access to effective treatment.

## Introduction

 Scabies is a highly contagious skin infestation caused by the *Sarcoptes scabiei* mite. In recent years, scabies have re-emerged as a public health concern, with increasing incidence rates reported in various regions worldwide.^1 2^ However, although many authors and clinicians complain about a worsening and increase in diagnoses, well-standardised data certifying the status are often missing.^3 4^ A similar trend has been complained about in Bologna, Italy, with several scabies cases reported to local healthcare authorities. This study aimed to show the temporal trends of scabies diagnoses at a hospital in Bologna, focusing on identifying significant changes in incidence over time.

## Methods

This retrospective observational study analysed data on scabies cases diagnosed at the Dermatological Emergency Department of S. Orsola Hospital in Bologna, Italy, from October 2013 to September 2024. At this hospital, all patients presenting with pruritic symptoms are referred from the general emergency department to the dermatology unit, where a confirmed diagnosis of scabies is established through dermoscopic or microscopic examination. Data were extracted from hospital electronic medical records (EMRs) using ICD-9 (International Classification of Diseases, Ninth Revision) codes specific to scabies diagnoses. Each case entry included monthly counts, discharge date (year and month), patient age and nationality, providing a comprehensive dataset to evaluate trends over time. Only first visits were included, excluding follow-up visits or post-therapy controls to avoid duplication.

Monthly scabies diagnoses were aggregated to identify both annual and seasonal trends. Temporal trends were analysed by calculating a 12-month rolling average, and the χ^2^ test was used to examine differences in case distribution by nationality. Linear regression was applied to assess the yearly increase in scabies incidence, with statistical significance determined by a p value threshold of 0.05. Additionally, seasonal variation was evaluated using the Kruskal-Wallis test to identify any statistically significant differences in case counts across months. All analyses were conducted using appropriate statistical software.

## Results

**Figure 1:**
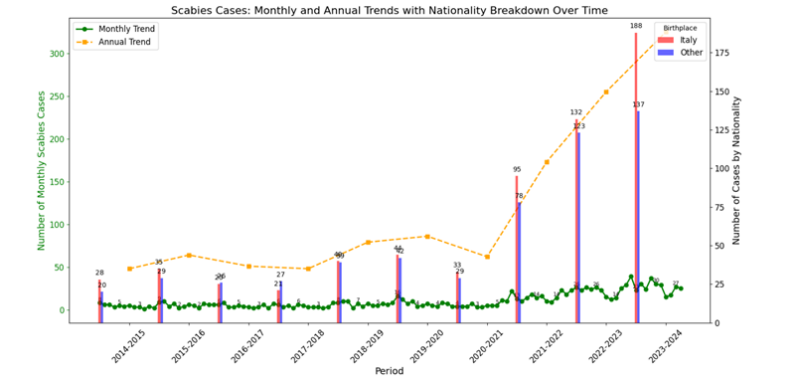
Trends in scabies cases over time showing monthly (green line) and annual (orange dashed line) case counts from 2014 to 2024. The chart includes a breakdown by patient nationality, with Italian-born individuals (red bars) and individuals from other nationalities (blue bars) highlighted for each reporting period. The data indicate a marked increase in scabies cases over the years, with notable peaks observed in the latest years.

During the study period, 1192 cases of scabies were diagnosed. The monthly distribution of cases is shown in figure 1, which presents the monthly trend of scabies cases and a 12-month rolling average to indicate the overall trend.

Monthly analyses using the χ^2^ test did not show a statistically significant difference in the distribution of scabies cases by nationality; however, when re-evaluated on an annual basis, a statistically significant difference, with an important difference between the first and the last year examined (χ^2^, p<0.01), highlighting a predominance of cases in the latest years among the Italian nationality residents.

A significant upward trend in scabies incidence was observed over the years, with an annual increase of approximately 70 cases (p=0.019). The regression analysis indicated that the year explained 48% of the variance in total scabies cases.

The analysis also showed seasonal variation in scabies cases, with certain months showing higher average cases than others. Specifically, March, April and February exhibited the highest mean case counts (40.4, 34.9 and 35.6, respectively), while July and August recorded the lowest averages (14.5 and 15.8, respectively) with a high significance (p<0.001).

## Discussion

The findings of this study indicate a marked increase in scabies cases in Bologna over the last decade, with significant annual growth and a clear seasonal pattern in case distribution. The results show that scabies cases peak during the winter months, specifically in February, March and April, and reach their lowest points in July and August. This seasonal variation aligns with previous research linking higher scabies transmission rates to colder months. This phenomenon may be driven by increased indoor crowding and closer physical contact during winter, which can facilitate the spread of scabies mites.^5^ The observed differences in scabies incidence between consecutive 12-month intervals suggest that this rise is not steady but increased suddenly in a wave-like pattern.

As the main referral hospital for the metropolitan area and the only one assisted by a dermatological department, this study’s findings likely provide a representative view of scabies incidence in Bologna. While milder cases treated outside the hospital may be under-represented, the large patient base, extended observation period and standardised diagnostic approach enhance the relevance of these findings for the entire urban setting.

Multiple interacting factors occurring concomitantly may result in this fluctuation. Social determinants, including population density, living conditions and migration patterns, might play a role in this fluctuation.

Among the potential factors contributing to the increase in scabies cases in Bologna is the recent rise in tourism.^6^ High levels of tourism can lead to overcrowded, low-quality accommodations, particularly for low-budget travellers but also for low-income citizens, who may lack proper hygiene and privacy. This phenomenon, often termed ‘overtourism,’ has contributed to disease transmission in crowded and inadequately sanitised environments. Such conditions likely create an environment conducive to scabies transmission, amplifying seasonal surges.

Without known drug resistance or demographic changes in the residential population,^7^ these findings suggest that environmental and social factors, including increased tourism, may influence scabies trends in Bologna. While the 2020–2021 spike in scabies diagnoses remains unexplained and is likely driven by factors beyond traveller fluxes, tourism in the city had begun increasing significantly a couple of years before the COVID-19 pandemic and resumed its upward trajectory in the 2 years following it. The interplay between local residents travelling abroad and short-term visitors to the city may have indirectly contributed to the rise in cases, increasing the risk of contagion for the resident population. Enhanced awareness, timely diagnosis and access to effective treatment are essential in managing this public health issue effectively.^8 9^

This study has several strengths and weaknesses. Its strengths include its long observation period, large case series and standardised disease coding in the EMR system, which ensures diagnostic consistency. However, as a retrospective study, it is subject to potential biases from historical data. Another limitation is the potential selection bias due to data sources: scabies cases come either through the emergency department or via direct referrals from general practitioners or other hospital units with urgent requests, which other outpatient clinics around the city can assist. While the exact proportion of those referrals remains unquantifiable, the emergency department pathway has remained consistent in access modalities over time, offering a stable benchmark for evaluating trends.

## Conclusions

The peak of diagnoses of scabies in Bologna highlights the need for enhanced public health.

While the absence of known drug resistance or significant demographic changes may limit some explanatory factors, the overall increase in cases could be influenced by various environmental and social factors. Among these, the growing influx of tourism and potential challenges associated with accommodation conditions may play a role, as suggested by some authors.^8 9^ These dynamics, alongside the observed seasonal variation, highlight the need for continuous monitoring and further research to better understand the drivers of scabies transmission. Continuous monitoring and targeted prevention and treatment strategies are recommended to address this public health issue effectively.

## Supplementary material

10.1136/sextrans-2024-056436 Abstract translation 1 This web only file has been produced by the BMJ Publishing Group from an electronic file supplied by the author(s) and has not been edited for content.
