# Supplementary material for: Scabies increasing incidence in Bologna from 2013 to 2024: a retrospective analysis
Source: Sex Transm Infect. 2025 Jan 29;101(4):e056436. doi: 10.1136/sextrans-2024-056436 (PMC12128787; doi:10.1136/sextrans-2024-056436)
Supplement: Abstract translation 1 [file sextrans-101-4-s001.docx]

Obiettivi: L'infestazione da scabbia, causata dall'acaro Sarcoptes scabiei, è recentemente emersa come una questione di salute pubblica nei paesi occidentali, con un'incidenza in aumento a livello globale. A Bologna, le autorità sanitarie locali riferiscono un incremento nelle diagnosi di infestazione da scabbia, sebbene i dati dettagliati di questa problematica siano poco noti e consultabili. Questo articolo mira ad analizzare le tendenze temporali dei casi di scabbia centralizzati e diagnosticati presso il Pronto Soccorso Dermatologico dell’Ospedale S. Orsola, con attenzione ai cambiamenti significativi delle diagnosi negli anni e alla loro variazione stagionale.

Metodi: È stato condotto uno studio osservazionale retrospettivo utilizzando i dati raccolti tra ottobre 2013 e settembre 2024, estratti dai registri ospedalieri tramite codici ICD-9. Le variabili includevano il numero mensile di casi, la data di dimissione, l’età del paziente e la nazionalità. Tutti i casi di prurito provenienti dal Pronto Soccorso sono stati valutati presso l’unità di dermatologia, con diagnosi confermata mediante esame dermoscopico o microscopico. Sono state incluse solo le prime visite, escludendo i controlli di follow-up o post-terapia per evitare duplicati. I casi mensili sono stati aggregati per identificare tendenze annuali e stagionali. La distribuzione delle nazionalità è stata analizzata con il test del chi-quadro (χ²), le modifiche nella tendenza annuale sono state esaminate tramite regressione lineare, mentre la variazione stagionale è stata valutata con il test di Kruskal-Wallis.

Risultati: Sono stati riscontrati in totale 1192 casi. La distribuzione delle nazionalità è rimasta stabile, senza differenze significative tra italiani e altre nazionalità. È stata osservata una tendenza significativa all’aumento dell’incidenza negli ultimi anni, con una variazione stagionale che mostra un picco di casi tra febbraio, marzo e aprile, e un minimo tra luglio e agosto.

Conclusioni: L’aumento dei casi di scabbia negli ultimi anni e i distinti picchi stagionali suggeriscono che fattori ambientali e sociali possano contribuire all’aumento dei casi trasmessi a Bologna. In assenza di importanti cambiamenti demografici e di chiare segnalazioni di resistenza farmacologica ai trattamenti in atto, riteniamo che fattori come l’aumento del turismo e le condizioni di alloggio subottimali potrebbero giocare un ruolo nella trasmissione dell’infestazione. Raccomandiamo un incremento nel monitoraggio della trasmissione da parte degli organi di salute pubblica, maggiore consapevolezza e interventi mirati per gestire efficacemente questa tendenza al rialzo.
